# “But it sounded confident”: the role of accuracy, tone, and disclaimers in users' medical decision-making

**DOI:** 10.3389/frai.2026.1785085

**Published:** 2026-07-29

**Authors:** Kerstin Denecke, Elia Gabarron

**Affiliations:** 1 Institute Patient-Centered Digital Health, Bern University of Applied Sciences, Bern, Switzerland; 2 Polibienestar Research Institute, University of Valencia, València, Spain

**Keywords:** artificial intelligence, chatbot, consumer health information, health informatics, trust

## Abstract

**Introduction:**

Artificial intelligence (AI)-powered chatbots are increasingly used in healthcare for applications ranging from symptom triage to lifestyle guidance. Their effectiveness depends not only on their ability to provide reliable information but also on users engaging with their advice while remaining aware of potential inaccuracies. This study investigated how users perceive AI-generated medical advice, with a particular focus on the roles of accuracy, conversational tone, and disclaimers.

**Methods:**

A survey was conducted with 115 participants who evaluated 15 chatbot scenarios representing five common health problems. Participants assessed chatbot responses varying in accuracy, tone, and the presence of disclaimers. Associations between these factors, user trust, willingness to seek second opinions, and participant characteristics (including age, health literacy, and prior chatbot use) were examined.

**Results:**

Accuracy emerged as the strongest predictor of user trust, although participants did not consistently identify inaccurate advice. Individual characteristics, including age, health literacy, and prior chatbot experience, showed no significant associations with trust. Differences in chatbot tone had little influence on user perceptions, with chatbots generally being viewed as confident regardless of phrasing. The effects of disclaimers on willingness to seek second opinions were mixed and varied across scenarios, suggesting that disclaimers alone may not effectively prevent over-reliance on chatbot advice. Open-ended responses highlighted trust in healthcare professionals and the importance of clearly communicating AI limitations.

**Discussion:**

This exploratory study, based on self-reported intentions in hypothetical scenarios, suggests that the accuracy of AI-generated medical advice is the primary determinant of user trust. Conversational tone appears to have limited influence, while disclaimers may not consistently promote appropriate caution. These findings indicate that healthcare chatbots should prioritize accuracy, clearly communicate their limitations, and encourage critical evaluation to achieve an appropriate balance between user trust and scepticism.

## 1 Introduction

The growing use of chatbots in healthcare has generated both enthusiasm and concern (Denecke and Gabarron, 2024). Artificial Intelligence (AI)-powered conversational agents or chatbots can now generate natural and personalized medical advice, ranging from symptom triage to lifestyle recommendations or even deliver elements of treatment (Vaidyam et al., 2019). Chatbots show promise in terms of expanding access to health information, triaging and diagnosing (Jungmann et al., 2019), as well as supporting patient self-management (Bickmore et al., 2013). However, their widespread adoption also introduces substantial risks: chatbot responses may be inaccurate, misleading or overly confident, and users may act on such advice without consulting a professional (Denecke et al., 2025). This risk could become even more serious when chatbots that are not specifically developed for medical purposes, such as ChatGPT, are used by patients. The risks for patient safety are high, as inappropriate trust or misplaced skepticism can lead to adverse health outcomes (Denecke et al., 2025).

A central challenge in this field is designing AI interactions that foster appropriate trust calibration. Users must feel confident enough in the chatbot to engage with it and consider its suggestions, but they must also remain critical enough to question, verify or seek professional confirmation when the advice is inaccurate or potentially harmful.

Previous studies started examining users' trust in automated and conversational systems. Studies on algorithmic decision support have shown that cues such as displays of confidence and explanations can affect perceptions of reliability. For instance, users tend to over-rely on algorithmic advice, especially when tasks are objective or unfamiliar, but may also shift rapidly to distrust upon noticing errors (Araujo et al., 2020).

Systematic reviews of conversational agents in healthcare-specific settings have emphasized that most studies to date prioritize usability, system architecture or general user satisfaction, often overlooking critical safety or effectiveness issues (Laranjo et al., 2018; Milne-Ives et al., 2020). They found moderate evidence of usability and effectiveness, but highlighted limitations in study design and the paucity of long-term safety assessments.

Nov et al. conducted a Turing test to analyse whether patients can distinguish chatbot answers from human answers, finding that ChatGPT's responses were weakly distinguishable and that patients appear to trust chatbots for addressing lower-risk health questions (Nov et al., 2023). Research on disclaimers and content warnings indicates that they may impact reliance and perceived accountability, albeit inconsistently or to a limited extent (Lermann and Kimmerle, 2025). However, little is known about whether chatbot users can identify unsafe medical guidance, how stylistic factors such as tone or confidence affect trust and intended actions, or whether content warnings meaningfully influence reliance. This knowledge gap is important because over-trust in chatbots (due to their use of confident tone, or display of confidence) may encourage risky self-management, while excessive skepticism could prevent people from taking advantage of these tools' potential benefits.

This study examines user responses to AI-generated medical advice in scenarios involving common symptoms of varying severity, such as chest pain, rashes, headaches accompanied by visual disturbances, heartburn and high fever in children. Our three objectives are:

- Investigate whether users can detect harmful or inaccurate medical advice in chatbot interactions,
- Examine how the tone, confidence, and presence of warnings affect user trust in chatbots and their intended actions, such as seeking further medical attention or modifying their behavior based on the advice provided,
- Identify design implications for the safe deployment of AI in health-related contexts, including guidelines for developing chatbots that prioritize user wellbeing and provide accurate, trustworthy information.

By addressing these objectives, this study aims to contribute to a deeper understanding of the complex interactions between users, chatbots, and healthcare systems, ultimately informing the development of safer, more effective, and more user-centered AI solutions in health-related contexts. We focus on three research questions:

- RQ1: how well can users detect accurate or inaccurate medical advice from AI-generated responses?
- RQ2: is the chatbot's tone (e.g., confident vs. tentative) associated with differences in user trust and reported willingness to act on advice?
- RQ3: are content disclaimers (e.g., “Not a medical professional”) associated with differences in users' reported likelihood to seek a second opinion?

This work has two key contributions. Firstly, we present empirical evidence of how individuals process and respond to potentially hazardous AI-generated medical advice. Secondly, we offer design guidelines for conversational agents in health-related settings, with a focus on tone, confidence and disclaimers. Our findings emphasize the dangers of false reassurance and the inadequacy of generic disclaimers, as well as the significance of interaction design in minimizing over-reliance on AI systems. Together, these insights contribute toward safer and more user-centered approaches to the deployment of chatbots in healthcare.

## 2 Methods

In the following, the methods applied to answer the research questions, are described.

### 2.1 Study design

To answer our research questions, we used a cross-sectional online survey approach. This study followed the CHERRIES guidelines (Checklist for Reporting Results of Internet E-Surveys, see Appendix 2) (Eysenbach, 2004). The study design was submitted to the ethics committee of Cantone Bern, which confirmed that no ethics approval was necessary (Req-2025-01071).

An online survey that included 15 scenarios was created. For creating these scenarios, we developed chatbot conversations for five health problems: chest pain, rash, headache with visual disturbance, heartburn, and child with high fever. We selected these five problems as we assume them to be understood by the participants easily and they can imagine to follow the instructions of the chatbot. They range from routine (heartburn, many rashes) to time-critical (chest pain, pediatric fever).

For each scenario three example interactions were generated by Genspark.ai using the Mixture of agents feature in the AI chat. Three aspects were varied: 1) accuracy (accurate or inaccurate chatbot response); 2) confidence (confident or tentative answer by chatbot); and 3) disclaimer (disclaimer added to the chatbot response or not). The classification of chatbot responses as “accurate” or “inaccurate” was defined by the authors based on their assessment against standard medical knowledge, not through external clinical validation. No chatbot-generated response was evaluated or verified by a clinician or compared with clinical guidelines. Readers should interpret “accuracy” in this study as author-labeled accuracy for the purpose of analyzing user responses, rather than clinically validated accuracy. The wording and strength of disclaimers varied across scenarios (see Appendix 1), as they were generated by the AI. For example, some disclaimers stated “This advice is not a substitute for professional medical opinion” while others used phrasing such as “I am an AI and cannot diagnose” or “Please consult a healthcare provider.” In this study, the disclaimer presents as a binary variable (present vs. absent).

The following prompt was used to create the scenarios: “Create distinct medical chatbot conversations for five scenarios, Chest Pain (potentially dangerous) and Rash (harmless) and Headache with Visual Disturbance and Recurring Heartburn and Child with High Fever, with variations in tone (confident vs. tentative), disclaimer presence or absence, and accuracy (statement is accurate or inaccurate). Store the results in an Excel sheet. Here is an example for “heartburn,” Accuracy: correct,” Confidence: confident,” Disclaimer: no”: user: I've had heartburn after eating for the past week. It's getting a bit worse each day. What should I do?” Chatbot: “This sounds like acid reflux. Try avoiding spicy or fatty foods and take an antacid or proton pump inhibitor like omeprazole. If symptoms continue, see a doctor.” Create three examples per each health problem. Ensure a balanced dataset among the three variables”.

The dataset comprised 10 correct statements and five incorrect statements from the chatbot. Seven answers had a disclaimer, and eight did not. Eight generated chatbot answers were formulated indicating confidence with the answer, and seven answers were formulated rather tentatively. The 15 scenarios were designed as illustrative vignettes rather than as a fully crossed experimental manipulation. The three features of interest (accuracy, tone, and disclaimer) were not independently and orthogonally varied across all combinations due to the limited number of scenarios per health problem. Consequently, the analyses should be interpreted as exploratory and descriptive (examining patterns and associations) rather than as causal estimates of independent or interaction effects. Scenarios were not randomized or counterbalanced. Consequently, potential order effects (including participant learning, fatigue, or carryover from earlier scenarios to later ones) cannot be ruled out. For example, repeated exposure to chatbot advice across sequential vignettes may have influenced participants' response strategies or criticality over time. The full list of scenarios and generated conversations, indicating their chatbot-generated accuracy, generated confidence, and use of disclaimer are presented in Appendix 1.

### 2.2 Recruitment and survey administration

The online survey was created in both German and English as Microsoft Forms Online Questionnaire and distributed via TestingTime.com, a voluntary participant recruitment platform. The translation was conducted manually by one author. Before its distribution, the survey was evaluated in terms of performance and usability. To do so, the authors accessed the questionnaire using various devices, an iPhone, a MacBook Air, and iPad in different bowsers (Firefox, Safari, Google Chrome). The survey has been uploaded to Zenodo: https://zenodo.org/records/20447857.

We aimed at recruiting 100 participants aged 18–65, of any gender, and with language skills in either German or English. This number was chosen for pragmatic reasons: as the platform was not yet used for a survey by the authors and to balance the costs, we chose the smallest possible number for online questionnaires available on that platform. The online survey was launched on August 30 and closed on August 31, 2025 after receiving more than 100 responses. The recruitment costs summed up to CHF 1,500.

Recruited participants were asked to provide their informed consent before participating in the study. They were then requested to indicate their country of residence, gender, age group, and if they have ever used a chatbot for medical advice. No personal or identifiable information was collected. To assess their self-perceived health literacy, participants ranked their confidence in understanding health information using a 5-item Likert scale ranging from “Extremely not confident” to “Extremely confident.” Participants were asked next to evaluate each of the 15 scenarios by responding to a series of statements on a 7-item Likert scale, ranging from “Strongly agree” to “Strongly disagree.” These statements assessed whether the provided advice was accurate, whether the chatbot sounded confident, whether participants would follow the advice, and whether they would seek a second opinion. All outcomes were self-reported intentions in response to hypothetical scenarios, not observed real-world behavior. Participants reported what they would do (e.g., “I would follow the advice”) rather than actually following or not following advice in a clinical situation. Participants were also allowed to provide qualitative feedback on what made them trust or distrust the advice. All closed questions were mandatory to be answered. The questionnaire could only be sent when all mandatory questions were answered. Each scenario with corresponding judgement was shown on a separate page. In total, the questionnaire comprised 19 pages. Participants were able to go back to questions and change their answer at any time. The recruitment platform did not allow for multiple participation in the same questionnaire. No consistency or completeness checks were performed on the responses.

### 2.3 Data analysis

The data were analyzed both quantitative and qualitatively. Statistical analyses were performed using SPSS version 28.0.1.0 for Mac (IBM Corp., Armonk, NY, USA). Only complete surveys were analyzed. Descriptive statistics were used to summarize data. To analyse the association between individual factors and participants' trust in the chatbot-generated medical advice, a linear mixed-effects model analysis was used. The dependent variable was participants' rating on trust in each of the 15 scenarios, measured on a 7-points Likert scale from “Strongly disagree” to “Strongly agree.” The Likert-scale outcomes were treated as continuous; this approach is common in mixed-effects modeling when response categories are seven or more (Norman, 2010; Sullivan and Artino, 2013). Fixed effects were age group, gender, chatbot use, health literacy, and accuracy of the chatbot provided content. Random intercepts were included for participants to account for repeated measures across the 15 scenarios, allowing for individual differences in baseline trust levels. The model did not include random intercepts for individual scenarios or clinical condition (e.g., chest pain, rash) because the small number of scenarios per condition (three per health problem) and the lack of full crossing across experimental features prevented stable estimation of scenario-level random effects. This represents a design limitation, as scenarios were treated as exchangeable replicates rather than nested within clinical conditions. The model estimated the variance attributable to individual differences and also the residual variance. Type III tests of fixed effects were used to assess the significance of predictors. Additionally, Wilcoxon tests were used to test the perception of chatbot confidence and willingness to follow advice according to its tone (i.e., confident or tentative) were used. This study included multiple exploratory, scenario-level hypothesis tests, such as comparisons between perceived confidence and willingness to follow advice across 15 scenarios, as well as comparisons of willingness to seek a second opinion in scenarios with and without disclaimers.

To explore any optional free-text information that participants could provide for each of the 15 scenarios we applied two language models. We used the Gemma-2 language model with 9 billion parameters and the Llama 3 language model with 8 billion parameters without human supervision to conduct a thematic analysis. This was implemented in Python using the Ollama framework API (https://ollama.com). The analysis was conducted locally on a MacBook Air with an Apple M2 chip and 24 GB of RAM. The free-text comments could be written in either English or German. This is the prompt that was used: “*You are an expert in qualitative research and thematic analysis. You will analyse survey data from free-text responses to the question: “What made you trust or distrust the chatbot's advice in this case?” Instructions: 1) Read all responses carefully; 2) Identify recurring themes, patterns, and underlying reasons for trust or distrust; 3) Group responses into coherent themes (e.g., accuracy of advice, clarity, tone, empathy, prior knowledge, lack of transparency). For each theme: 1) Provide a concise label; 2) Write a short description; 3) Include representative example quotes from the data (if available). Summarize overall insights, highlighting the main drivers of trust and distrust. Output format: themes table/list with labels, descriptions, and examples. Summary section: key findings, distinctions between trust and distrust, and implications*.” We report on the themes identified by each of the language model in the result section.

## 3 Results

### 3.1 Participants

A total of 119 surveys were started, but only 115 were completed (40 in English, 75 in German). The sociodemographic characteristics of the study participants are presented in Table 1.

**Table 1:** Sociodemographic characteristics of the respondents (*n* = 115).

Sociodemographic characteristics	Frequency (percentage)
Country of residence	
Germany	40 (34.8%)
United Kingdom	27 (23.5%)
Switzerland	24 (20.9%)
Spain	2 (1.7%)
France	2 (1.7%)
Italy	2 (1.7%)
The Netherlands	2 (1.7%)
Austria	1 (0.9%)
Sweden	1 (0.9%)
Canada	1 (0.9%)
Not specified	13 (11.3%)
Gender	
Female	60 (52.2%)
Male	53 (46.1%)
Non-binary	1 (0.9%)
Prefer not to say	1 (0.9%)
Age group	
18–24	14 (12.2%)
25–34	33 (28.7%)
35–44	24 (20.9%)
45–54	24 (20.9%)
55+	19 (16.5%)
Not specified	1 (0.9%)

Sixty two among the 115 participants (53.9%) indicated that they have never used a chatbot for medical advice: 52 (45.2%) said they have used a chatbot for medical advice, and one person indicated that they did not know what a chatbot is. Regarding self-perceived health literacy, nine participants (7.8%) reported feeling extremely confident in understanding health information independently, 44 (38.3%) reported being very confident, and 56 (48.7%) described themselves as somewhat confident. In contrast, two participants (1.7%) reported being somewhat not confident, and four participants (3.5%) indicated they were extremely not confident.

### 3.2 Patterns in users' ability to detect accurate or inaccurate medical advice

When we analyzed the level of agreement on the accuracy of the different scenarios, we found that participants largely agreed that accurate chatbot-generated advice was indeed accurate, with agreement levels above 65% and reaching over 90% in some scenarios (i.e., scenario 6), see Table 2. In contrast, inaccurate advice received higher disagreement rates, often above 40% and up to 76.5% (i.e., scenario 11) though some inaccurate scenarios still saw notable agreement, suggesting occasional difficulty in detecting incorrect advice.

**Table 2:** Participants responses to the statement “I think the advice is accurate”.

Accuracy of chatbot generated advice	Disagreement^a^ frequency (percentage)	Neutral frequency (percentage)	Agreement^b^ frequency (percentage)
Accurate scenarios			
1-Chest pain	14 (12.2%)	10 (8.7%)	91 (79.1%)
3-Chest pain	4 (3.5%)	11 (9.6%)	100 (87.0%)
4-Rash	21 (18.3%)	19 (16.5%)	75 (65.2%)
6-Rash	6 (5.2%)	10 (8.7%)	99 (86.1%)
7-Headache with visual disturbance	11 (9.6%)	12 (10.4%)	92 (80.0%)
9-Headache with visual disturbance	18 (15.7%)	17 (14.8%)	80 (69.6%)
10-Heartburn	15 (13.0%)	16 (13.9%)	84 (73.0%)
12-Heartburn	13 (11.3%)	15 (13.0%)	87 (75.7%)
13-Child with high fever	20 (17.4%)	9 (7.8%)	86 (74.8%)
15-Child with high fever	34 (29.6%)	10 (8.7%)	71 (61.7%)
Inaccurate scenarios			
2-Chest pain	48 (41.7%)	36 (31.3%)	31 (27.0%)
5-Rash	67 (58.3%)	23 (20.0%)	25 (21.7%)
8-Headache with visual disturbance	48 (41.7%)	26 (22.6%)	41 (35.7%)
11-Heartburn	88 (76.5%)	14 (12.2%)	13 (11.3%)
14-Child with high fever	82 (71.3%)	13 (11.3%)	20 (17.4%)

^a^Disagreement includes ratings strongly disagree, disagree, and somewhat disagree;

^b^Agreement includes ratings somewhat agree, agree, and strongly agree.

### 3.3 User factors associated with trust in chatbot advice

The mixed-effects analysis examined the impact of individual factors and chatbot content accuracy on participants' trust in the advice (Table 3). Age, treated as a continuous covariate, was not significantly associated with trust (*p* = 0.333). None of the gender categories differed significantly from the reference group. Regarding chatbot usage, participants who indicated they have never used a chatbot for medical advice reported slightly higher trust, although this difference was not statistically significant compared to those who had used it. For perceived health literacy, treated as a continuous covariate, there was no significant association with trust. Accuracy was strongly associated with trust: inaccurate advice corresponded to substantially lower trust ratings compared to accurate advice (*p* < 0.001).

**Table 3:** Estimates of fixed effects from a mixed-effects model predicting trust in chatbot medical advice.

Coefficient	b (95% CI)	Sign.
Intercept	5.069 (3.449 to 6.689)	< 0.001
Age (continuous)	−0.057 (−0.174 to 0.059)	0.333
Gender		0.201
Gender female vs. reference	0.470 (−1.137 to 2.077)	
Gender male vs. reference	0.607 (−0.991 to 2.206)	
Gender non-binary vs. reference	−0.669 (−2.870 to 1.532)	
Chatbot use		0.094
No previous chatbot use vs. reference	0.254 (−0.044 to 0.551)	
Health literacy (continuous)	−0.114 (−0.298 to 0.071)	0.226
True accuracy		< 0.001
Inaccurate vs. reference	−2.006 (2.136 to −1.876)	

Reference categories: gender: “prefer not to say”; Chatbot_Use: “has used chatbot for medical advice”; True_Accuracy: “accurate advice”.

### 3.4 Model diagnostics

Model assumptions were assessed using residual diagnostics. The Shapiro-Wilk test on standardized residuals was significant (*W* = 0.976, df = 1,686, *p* < 0.001). Visual inspection of the Q–Q plot showed a slight inverted U-shaped pattern. Standardized residuals ranged approximately from −3 to +3. A scatterplot of standardized residuals against predicted values showed no evident curvature or funnel-shaped pattern.

### 3.5 Association between chatbot's tone (e.g., confident vs. tentative) and user responses

Participants generally rated the chatbot as sounding confident independently if the generated content was “confident” or “tentative” (see Table 4). However, in scenarios 5, 11, 13, and 14 for confident responses, and in scenarios 8, 9, and 15 for tentative responses, the ratings for willingness to follow the advice provided by the respondents were significantly lower than ratings of chatbot confidence (*p* < 0.05).

**Table 4:** Comparison of the perceived chatbot confidence and willingness to follow advice.

Generated confidence	I think the chatbot sounds confident in what it says mean (std. deviation)	I would follow the advice mean (std. deviation)	Z Wilcoxon signed rank test	*P* (Bonferroni-adjusted)
Confident				
1-Chest pain	5.23 (1.504)	5.23 (1.552)	−1.022, *p* = 0.307	1.000
4-Rash	4.82 (1.508)	4.59 (1.544)	−1.616, *p* = 0.106	1.000
5-Rash	4.00 (1.816)	2.87 (1.553)	−5.872, *p* < 0.001	< 0.015
7-Headache with visual disturbance	5.43 (1.445)	5.26 (1.390)	−1,657, *p* = 0.097	1.000
10-Heartburn	5.63 (1.033)	5.49 (1.059)	−2.064, *p* = 0.039	0.585
11-Heartburn	4.00 (1.864)	2.44 (1.390)	−6.436, *p* < 0.001	< 0.015
13-Child with high fever	5.84 (1.005)	4.95 (1.756)	−5.414, *p* < 0.001	< 0.015
14-Child with high fever	4.04 (1.818)	2.54 (1.546)	−6.493, *p* < 0.001	< 0.015
Tentative				
2-Chest pain	3.92 (1.628)	3.75 (1.498)	−1.370, *p* = 0.171	1.000
3-Chest pain	5.40 (1.191)	5.58 (1.124)	−1.698, *p* = 0.089	1.000
6-Rash	5.71 (1.262)	5.94 (0.786)	−0.357, *p* = 0.721	1.000
8-Headache with visual disturbance	4.27 (1.677)	3.71 (1.626)	−3.387, *p* < 0.001	< 0.015
9-Headache with visual disturbance	5.09 (1.367)	4.88 (1.421)	−2.321, *p* = 0.020	0.300
12-Heartburn	5.54 (1.044)	5.10 (1.498)	−1.824, *p* = 0.068	1.000
15-Child with high fever	4.90 (1.595)	4.30 (1.681)	−4.750, *p* < 0.001	< 0.015

Bonferroni correction applied to 15 comparisons (α = 0.05/150 = 0.0033).

Adjusted p-values = p × 15 (capped at 1.000).

### 3.6 Willingness to seek second opinions: comparison of scenarios with and without disclaimers

**Figure 1:**
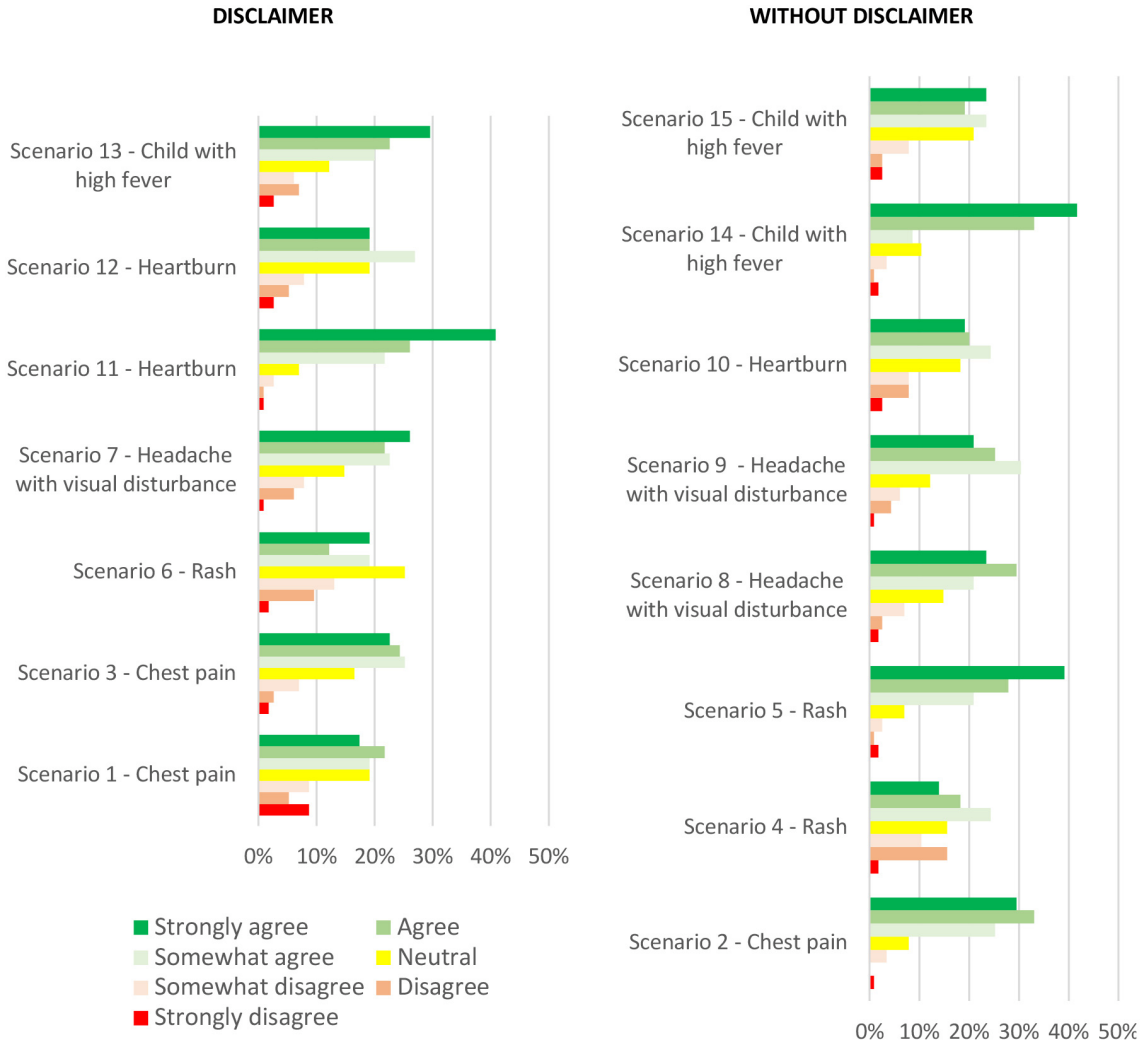
Percentage of agreement with the statement “I would seek a second opinion after reading this advice” by presence or absence of a chatbot disclaimer.

Figure 1 shows the percentage of agreement to the statement “I would seek second opinion after reading this advice” according to the presence or absence of a disclaimer on the advice provided by the chatbot.

Across the seven scenarios presented that included a disclaimer, the proportion of participants who expressed some level of agreement (“somewhat agree,” “agree” or “strongly agree”) with the statement “I would seek a second opinion after reading this advice” varied notably. Agreement was lowest in Scenario 6, where only 50.4% indicated agreement, and highest in Scenario 11, with 88.7% of participants agreeing. In Scenarios 1, 3, 7, 12, and 13, agreement levels ranged from 61.9% to 78.3%, while neutral responses ranged from 6.9% in Scenario 11 to 25.2% in Scenario 6. Disagreement (“somewhat disagree,” “disagree” or “strongly disagree”) was most prevalent in Scenario 6 (24.4%) and least common in Scenario 11 (4.4%).

In the eight scenarios where chatbot advice was provided without a disclaimer, the proportion of participants indicating agreement (“somewhat agree,” “agree” or “strongly agree”) with willing to seek a second opinion ranged from 40.9% in Scenario 14 to 88.7% in Scenario 5. Most scenarios showed a majority of participants willing to seek a second opinion, with particularly high agreement in Scenarios 5 (87.8%), 2 (87.8%), and 8 (73.9%). Neutral responses were generally low, ranging from 6.9% in Scenario 5 to 20.9% in Scenario 15, while disagreement was minimal across all scenarios, never exceeding 15.7%.

### 3.7 Additional information provided by the participants

In the exploratory analysis of participants' optional written information conducted with Llama3 and Gemma2 without human supervision, several overarching themes were identified.

Llama3 identified seven main themes: 1) Concerns about seeking medical attention (Respondents emphasized the importance of consulting a healthcare professional, particularly if symptoms persisted or worsened); 2) Skepticism about self-diagnosis (Participants highlighted the risks of relying solely on chatbots or online resources and stressed the value of professional assessment to avoid misdiagnosis); 3) Urgency and emergency situations (Respondents underlined the need for rapid intervention in cases of potentially life-threatening symptoms); 4) Importance of follow-up questions (Many participants noted that chatbot responses lacked sufficient contextual information, emphasizing the importance of asking clarifying questions to improve accuracy); 5) Trust in healthcare professionals (A recurring theme was the strong reliance on medical expertise, with participants expressing greater confidence in professional advice compared to chatbot guidance); 6) Caution and hesitancy (Some participants expressed uncertainty regarding symptom severity or risks); and 7) Education and awareness (Respondents pointed to the importance of health education and accessible resources in enabling informed decision-making).

Gemma2 identified four main themes: 1) User trust and perception (Participants were particularly sensitive to chatbot responses that used uncertain phrasing (e.g., “probably,” “maybe”), which reduced confidence. While many acknowledged AI's limitations, they emphasized the need for certainty in health matters and often sought validation from healthcare professionals); 2) Desired information from AI (Users expressed a preference for clear explanations and actionable next steps, such as when to seek medical care or what home measures to take. They valued responses where the AI asked follow-up questions to better understand their situation); 3) Areas for improvement (Participants suggested that chatbots should more explicitly acknowledge their limitations, particularly in serious cases, and strongly encourage seeking medical care when warranted. They also proposed linking to reliable resources to support informed decision-making); and 4) Ethical considerations (Respondents stressed the importance of transparency about interacting with AI rather than a human, noted concerns about potential algorithmic bias, and highlighted the need to safeguard health information).

It can be recognized that Llama3 and Gemma2 generated different thematic structures (seven vs. four themes). However, there was substantial overlap in the key issues identified from the free text comments. Both models highlighted the participants' preference for or consulting healthcare professionals, concerns regarding the limitations and uncertainty of AI-generated advice, the importance of seeking medical attention when appropriate, and the value of follow-up questions and context-specific information. However, the models' thematic clusters differ in their level of abstraction. Llama3 provided a more granular and descriptive thematic structure, separating issues such as trust in healthcare professionals, skepticism toward self-diagnosis, urgency of symptoms, caution, and health education into distinct themes. In contrast, Gemma2 consolidated several of these concerns into broader categories such as “User Trust and Perception” and “Desired Information from AI.” Gemma2 also generated themes related to “Areas for Improvement” and “Ethical Considerations,” including transparency, bias, and privacy, which reflect a more interpretive perspective on the content of the original comments. The combination of both results thus provide a more complete view on the free text comments.

## 4 Discussion

### 4.1 Summary of findings

This study assessed how users respond to AI-generated medical advice in various symptom scenarios. The focus was on their ability to detect accuracies and inaccuracies in generated health advice, depending on whether the generated text sounded confident or tentative, and whether or not disclaimers were present. We found that participants were generally able to recognize accurate advice as trustworthy and often—though not always—identified inaccurate advice as incorrect. The mixed effects analysis showed that trust in chatbot advice was not statistically associated with age or gender. Trust was slightly higher among those who had never used a chatbot for medical advice or reported perceived low health literacy. The chatbot accuracy had the strongest effect, with inaccurate advice significantly reducing trust. The chatbot tone showed limited association with user perceptions: participants tended to perceive the chatbot as confident, regardless of whether the responses were framed as “confident” or “tentative,” although their willingness to follow the advice sometimes diverged from these perceptions. Disclaimers showed mixed and scenario-dependent patterns in relation to willingness to seek a second opinion, suggesting that they are not reliable safeguards against over-reliance. An exploratory analysis of the optional feedback highlighted themes such as the participants' caution about self-diagnosis, their trust in healthcare professionals, and the need for clearer, and more transparent AI guidance.

### 4.2 Detecting inaccurate advice (RQ1)

Participants showed a relatively strong consensus when evaluating accurate responses. Disagreement with inaccurate advice was more variable: in some scenarios elicited high levels of skepticism, while others saw notable agreement. This indicates that users did not always recognize unsafe guidance, which could point to a possible automation bias. Automation bias occurs when individuals over-rely on automated systems and assume that the provided information by the AI system is correct simply because it comes from a technological source (Romeo and Conti, 2025; Rice, 2009; Goddard et al., 2012). In this study, some participants may have assumed that the chatbot's advice was trustworthy without evaluating its accuracy, which could potentially translate into unsafe decisions in real-world scenarios. However, several participants pointed to the risks of relying solely on chatbots or online resources, and emphasized the importance of trusting healthcare professionals, highlighting that they generally place greater confidence in advice provided by qualified clinicians than in chatbot-generated guidance.

Previous research studying the effect of individuals characteristics in detecting medical advice inaccuracies provided by AI has found that female clinicians tend to be more critical toward AI-generated health content (Kücking et al., 2024) however age did not have a significant effect. In contrast, in our study, gender was not a factor that significantly influenced trust, and age also showed no significant association. While a previous study focused on clinicians (Kücking et al., 2024), our study may involve a general population, although we cannot confirm this, as data on participants' educational level or professional background were not collected.

Findings from this study also suggest that individuals' use of chatbots may influence levels of trust in chatbot advice. This aligns with the concerns raised in reviews of medical chatbots, which emphasize the potential risks of false reassurance and unsafe advice when users lack the expertise to validate system outputs (Laranjo et al., 2018). It also agrees with the theme of education and awareness, where participants highlighted that access to reliable health information empowers users to make more informed decisions.

We can recognize a variation depending on the scenario which may indicate the importance of context and prior knowledge. Conditions such as chest pain or pediatric fever are widely recognized as high-risk, making inaccurate reassurance easier to detect. In contrast, ambiguous conditions such as rashes or heartburn leave more room for misclassification. This suggests that users' ability to detect inaccuracy varies, which reinforces the idea that safe chatbot design cannot depend on user vigilance alone. Another limitation of the current design is the absence of a comparator condition involving clinician- or guideline-based advice. Including such a comparator would have helped determine whether the observed difficulties in detecting inaccuracies are specific to AI-generated advice or reflect broader challenges in evaluating medical information regardless of source. Future research could study these aspects in more depth.

### 4.3 Tone and user trust (RQ2)

We explored whether the chatbots' tone (confident or tentative) would be associated with differences in perceptions of trust and willingness to follow advice, but the results revealed a more nuanced pattern. Participants consistently rated chatbots as “confident”, regardless of whether the response was linguistically framed as confident (e.g., Scenario 11: “*That's nothing to worry about—just ignore it. Heartburn is never dangerous. However, this advice is not a substitute for a professional medical opinion”*) or tentative (e.g., Scenario 8 “*It's probably just eye strain or dehydration. Try resting and drinking more water”*). This suggests that wording style alone may not effectively communicate uncertainty to users. However, there were scenarios in which the willingness to follow advice was significantly lower than the perceived confidence, indicating that users sometimes separate perceived authority from behavioral intention. For example, in cases involving potentially serious or acute health issues (e.g., Child with high fever), a statistically significant discrepancy was found between participants perceiving the chatbot's advice as confident and their willingness to follow it. In such cases, participants might have balanced risks and benefits, as reported in previous research (Hipgrave et al., 2025), which could indicate their awareness of the limitations of AI advice. This dissociation may indicate a cognitive gap: while tone may shape initial impressions, practical decision-making can be influenced by other factors such as perceived risk (Kerstan et al., 2024), previous health knowledge (Biro et al., 2023; Jin et al., 2025), or past experience with chatbots (Wu et al., 2024). From the perspective of the dual-process theories, this discrepancy could be interpreted as a conflict between System 1 (fast and intuitive processing) and System 2 (slow and analytical processing) (Evans, 2008; Evans and Stanovich, 2013). The chatbot's confident tone of the chatbot may engage System 1, and generating an initial impression of trustworthiness. However, when considering a potential high-risk scenario (e.g., involving a child), factors like perceived risk may activate System 2, prompting the individual to re-evaluate the scenario, resulting in greater skepticism and reduced willingness to follow the chatbot's advice.

These findings contribute to the existing literature on how tone and framing can influence perceived credibility (Flusberg et al., 2024; Okoso et al., 2025; Qin et al., 2024), although these effects are often moderated by the plausibility of the content and the context of the task. Specifically in healthcare, prior studies have shown that users value clear, directive communication highly, which may explain why tentative phrasing did not reliably reduce perceptions of confidence (Bickmore et al., 2018). Open-ended feedback provided by the participants support this interpretation, as they reported that the use of uncertain expressions, such as “probably” or “maybe” reduced their confidence in the chatbot provided advice.

Our results therefore highlight a design challenge: tone alone is not a reliable means of calibrating trust, and communicating uncertainty may require explicit indicators (e.g., probability ranges or structured risk statements) rather than subtle linguistic cues. Future research could investigate the use of different ways to show chatbots' uncertainty, such as using visual risk indicators or confidence meters that could help users better understand the limits of the AI system. These approaches may support users in recognizing the limitations of AI systems, reduce automation bias, and promote a more deliberated decision-making (System 2).

### 4.4 Disclaimers and second opinion (RQ3)

In line with previous research recommending including explicit disclaimers in chatbots to minimize the risk of misleading users (Choudhury and Shamszare, 2023), more recent publications found that about 70%−100% of chatbot responses included disclaimers about health advice, and that these disclaimers helped users recognize that the information provided was not a substitute for professional medical advice (Atahan et al., 2025; Scaff et al., 2025). In this study, however, the presence of disclaimers showed only a modest and inconsistent association with participants' stated willingness to seek a second opinion, and this association varied depending on the scenario. In some scenarios, for example, in low perceived risk scenarios (e.g., Scenario 6 - Rash), the use of a disclaimer did not increase the willingness to seek a second opinion compared to scenarios without disclaimers, suggesting that the impact of disclaimers on human behavior may be limited. These findings could be explained by cognitive factors, such as users' over-reliance on the chatbot or an automation bias (Romeo and Conti, 2025; Rice, 2009; Goddard et al., 2012), where disclaimers may be overlooked. On the other hand, open-ended feedback from participants indicated that more explicit acknowledgment of the chatbot's limitations was desirable, together with stronger recommendations to seek medical care, particularly in serious cases. These findings might indicate that the simple use of disclaimers may not be sufficient to guide user safely, and as suggested in a previous publication (Moëll and Sand Aronsson, 2025), a combination of explicit guidance, contextual cues, and clear risk communication, among other strategies, may be needed to promote users' safer decision-making and reduce harm. However, because disclaimer wording varied across scenarios, these findings should be interpreted as exploratory observations about heterogeneous set of disclaimers rather than as test of disclaimer presence vs. absence.

The fact that chatbots' disclaimers sometimes increase users' caution but sometimes have little effect, making them unreliable as a safety mechanism, is particularly concerning in healthcare-related contexts. Furthermore, recent work has documented that many commercial chatbots have removed medical disclaimers altogether despite a lack of evidence regarding their protective value (O'Donnel, 2025). Our findings suggest that, if used, disclaimers should be contextualized to the risk of the presenting symptom and ideally paired with actionable prompts (e.g., Scenario 1: “*This could be a heart attack. Please call emergency services or go to the nearest ER immediately. This advice does not replace medical care—chest pain can be life-threatening”*). When using disclaimers, dual-process theories should be also considered (Evans, 2008; Evans and Stanovich, 2013), where the use of risk-framed language may engage more analytical thinking (System 2), and thereby reduce automatic trust in AI (System 1). Future research could investigate the influence of contextualized disclaimers on users' behavior, and how cognitive factors such as automation bias or dual process thinking affect users' responses to disclaimers, and whether their impact differs between low and high perceived risk scenarios.

### 4.5 Implications for chatbot conversation design

**Figure 2:**
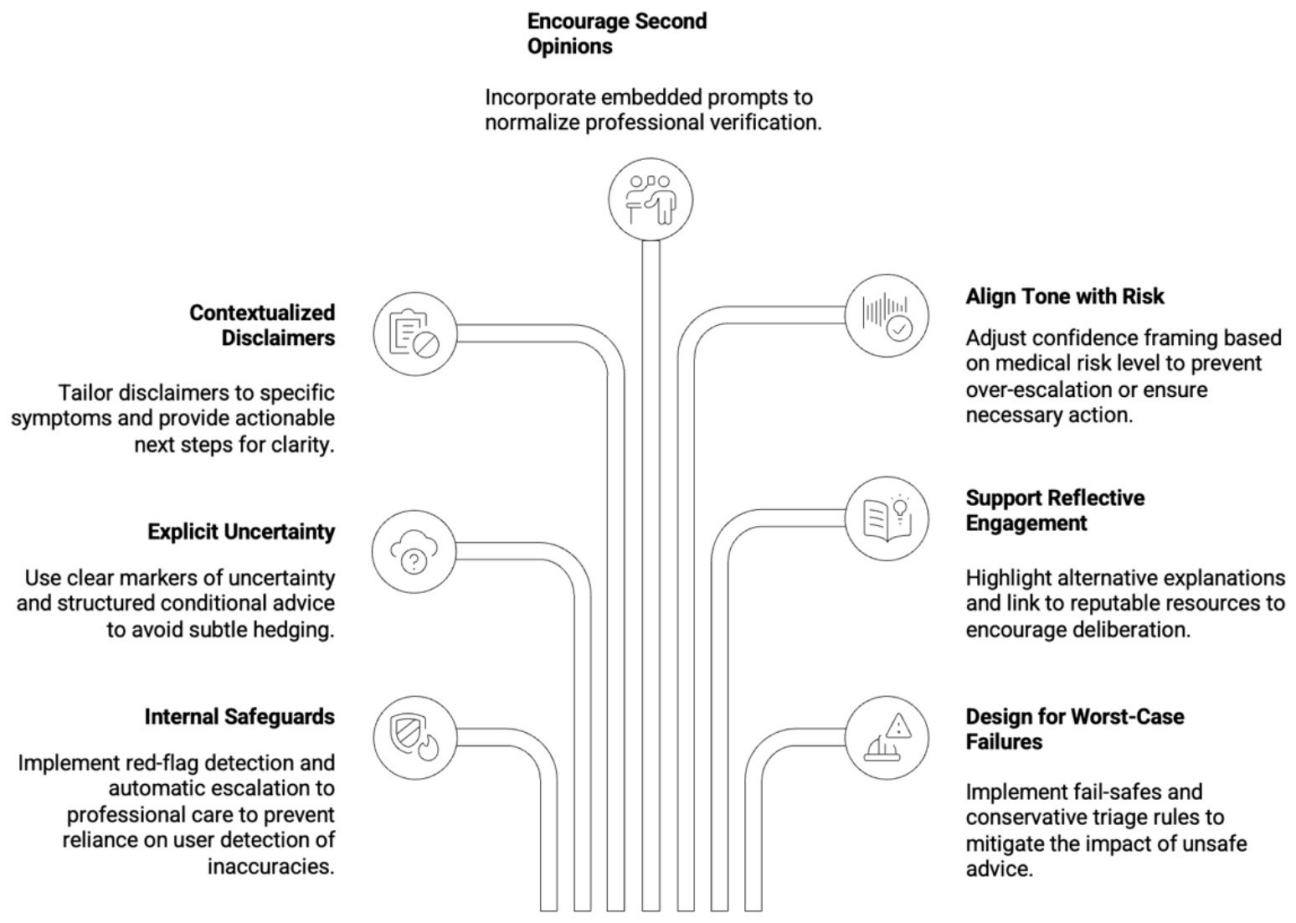
Practical design guidelines (figure generated from manually written text using napkin.ai).

Across the three RQs, a consistent theme emerges: users cannot be expected to reliably detect inaccuracy, subtle cues like tone are insufficient to calibrate trust, and disclaimers alone are not a dependable safeguard. This has several implications for chatbot conversation design, shown in Figure 2. Chatbots must integrate more robust safeguards, including:

- Explicit and standardized uncertainty communication (beyond linguistic hedging),
- Structured prompts to encourage second opinions, especially for high-risk symptoms,
- Automated escalation for red-flag scenarios, and
- Contextualized, symptom-specific warnings rather than generic disclaimers.

### 4.6 Limitations and future work

This study has several limitations that should be considered when interpreting the findings. Information about participants' educational background was not collected, despite this potentially being an important factor influencing their ability to critically evaluate chatbot advice. The sample size of around 100 participants raises questions about its representativeness, particularly when drawing conclusions about diverse populations. Additionally, the recruitment method introduced potential bias as participants were drawn from an online crowdsourcing platform and were therefore likely to possess moderate levels of digital literacy. This may not reflect the broader population of health service users. The participant pool was geographically limited, with most respondents originating from three European countries, which restricts the generalizability of the findings to other cultural and healthcare contexts. Since the survey was distributed through Testingtime.com, data on view rates and participation rates were not available and therefore could not be analyzed.

The main themes reported by participants through open-ended fields were analyzed using an exploratory approach with large language models. While large language models can help identify themes in large datasets (Fischer and Biemann, 2024), these analyses were not contrasted with human coding (Prescott et al., 2024) which represents a significant limitation and could have introduced bias in the interpretation of qualitative data. The models produced a differing theme structure which could have been avoided by first defining the codes manually and let the model assign the codes. Such procedure would have been more close to a manually conducted thematic analysis. However, the free text comments were not supposed to be a major source of results, but only additional reflections given the subjective nature of these comments. Prior research has shown that LLM outputs can be sensitive to prompt wording, contextual framing, and the tone of the input, which may introduce response or confirmation biases (Schroeder et al., 2025). In consequence, the themes generated from the participants' comments may partly reflect model-specific interpretive tendencies rather than solely the underlying participant responses. Although the use of two different models provided some opportunity to assess consistency across outputs, the observed differences in thematic structure indicate that LLM-generated qualitative analyses may vary depending on the model employed. Therefore, the identified themes should be regarded as exploratory and interpreted with appropriate caution.

The scenario design was not fully factorial: accuracy, tone, and disclaimer conditions were not independently crossed across all 15 vignettes due to the limited number of scenarios per health problem. Consequently, we cannot estimate independent or interaction effects causally, and our findings should be interpreted as exploratory associations. Further, we did not check whether the tone differing in the vignettes which could have introduce bias. Additionally, our mixed-effects model included random intercepts for participants but not for scenarios or clinical conditions, reflecting the pragmatic constraints of the design. Scenarios were treated as exchangeable replicates rather than nested within health problems. Future studies should employ crossed designs with larger scenario sets, enabling three-level hierarchical models (participants × scenarios × conditions) and more precise estimation of variance components. Scenarios were presented in a fixed order rather than randomized or counterbalanced, which raises the possibility of order effects, including learning (participants becoming more adept at detecting inaccuracies over time), fatigue (reduced attention or engagement across 15 sequential vignettes), or carryover effects (judgements on earlier scenarios influencing responses to later ones). These factors may have influenced scenario-level findings, and our results should be interpreted with this limitation in mind. Future studies should employ randomization or counterbalancing to mitigate such order effects. Additionally, our choice of “Prefer not to say” as the reference category for gender was suboptimal for interpreting the association between gender and trust in chatbot advice. The classification of chatbot responses as “accurate” or “inaccurate” lacked clinical validation. This limits the construct validity of the accuracy variable and may affect the generalizability of findings to real-world clinical contexts. Future research should validate chatbot-generated advice against clinician panels or established clinical guidelines to strengthen construct validity and clinical relevance. The scenario-level hypothesis tests were not adjusted for multiple comparisons. Given the modest sample size and the hypothesis-generating nature of the study, we prioritized statistical power (i.e., reducing the risk of Type II error, or false negatives) over strict control of Type I error. Readers should interpret individual *p*-values with caution, as the risk of false-positive findings increases with the number of comparisons conducted. These analyses should be considered exploratory and hypothesis-generating, rather than confirmatory. Our study did not include a comparator condition such as clinician-authored advice or guideline-consistent responses. Without such a comparator, it is difficult to determine whether the observed patterns are specific to AI-generated advice or reflect general responses to medical advice regardless of source. This study measured self-reported intentions in response to hypothetical vignettes rather than observed real-world behavior. Participants indicated what they would do (e.g., follow advice, seek a second opinion) in imagined scenarios, which may differ from actual behavior in real clinical situations due to factors such as time pressure, emotional state, social context, or perceived consequences. Therefore, our findings should not be directly extrapolated to real-world patient safety or clinical decision-making without further validation using behavioral or naturalistic study designs. Finally, the disclaimers used across scenarios were not standardized in wording or strength as they were AI-generated responses produced naturally varying disclaimer language. Our comparison of disclaimer presence vs. absence may confound the effect of disclaimers with differences in their phrasing, specificity, or perceived urgency. Future studies should standardize disclaimer language or explicitly distinguish between disclaimer types (e.g., generic vs. specific, weak vs. strong) to enable clearer interpretation.

## 5 Conclusions

This study assessed how users respond to AI-generated medical advice in various health scenarios. Participants were generally able to recognize accurate advice and often, but not always, identified inaccurate advice, indicating that while AI-generated health information can be partially trusted, errors may go unnoticed, particularly for ambiguous or low perceived-risk conditions. The chatbot's confident or tentative phrasing had limited impact on user perceptions, as participants generally rated the chatbot as confident regardless of tone. While disclaimers modestly influenced users' willingness to seek a second opinion, their effect was highly scenario dependent. This highlights that subtle linguistic cues alone may be insufficient to communicate uncertainty to non-specialist users and that simple disclaimers cannot be relied upon to prevent over-reliance, especially in high-risk situations. Users' practical decisions could be influenced by their perceived confidence in chatbot advice, perceived risk, prior knowledge, and past experiences; these discrepancies can be contextualized within automation bias and dual-process thinking.

These findings have important design implications. Chatbot design must move toward stronger, standardized mechanisms for communicating uncertainty, guiding users to seek second opinions, and escalating high-risk situations. These measures can better protect users by ensuring safety-critical health information is conveyed clearly, contextually, and with appropriate safeguards.

## Data Availability

The original contributions presented in the study are included in the article/Supplementary material, further inquiries can be directed to the corresponding author. Appendix 1. List of the 15 generated scenarios by Genspark.ai Appendix 2. Checklist for Reporting Results of Internet E-Surveys (CHERRIES) The questionnaire has been uploaded to Zenodo: https://zenodo.org/records/20447857.
