# Supplementary material for: “But it sounded confident”: the role of accuracy, tone, and disclaimers in users' medical decision-making
Source: Front Artif Intell. 2026 Jul 29;9:1785085. doi: 10.3389/frai.2026.1785085 (PMC13462413; doi:10.3389/frai.2026.1785085)
Supplement: Supplementary file 1 [file Data_Sheet_1.pdf]

Study on Healthcare Chatbots / Studie zu Chatbots im Gesundheitswesen

* Erforderlich

Welcome! WIllkommen!

1. Welcome!
   This survey is part of a research study investigating how people interpret advice from Artificial Intelligence (AI)-powered chatbots in healthcare settings. You will read a few simulated conversations between a user and an AI chatbot and answer questions about each one.
   Your participation is voluntary and your responses are anonymous. Some scenarios may involve sensitive health topics. This survey takes about 10 minutes to complete.
   With sending your answers you consent to participate in the study.

   Willkommen!
   Diese Umfrage ist Teil einer Forschungsstudie, die untersucht, wie Menschen Ratschläge von KI-gestützten Chatbots im Gesundheitswesen interpretieren. Sie lesen einige simulierte Gespräche zwischen einer Person und einem Chatbot und beantworten dazu Fragen.
   Ihre Teilnahme ist freiwillig und Ihre Antworten bleiben anonym. Einige Szenarien können sensible

2. Would you like to answer the survey in English or German? / Möchten Sie den Fragenbogen auf Englisch oder Deutsch beantworten?

   ◯ English / Englisch

   ◯ German / Deutsch

Demographics

3. What is your age? *

- ◯ Under 18
- ◯ 18-24
- ◯ 25-34
- ◯ 35-44
- ◯ 45-54
- ◯ 55+

4. What is your gender? *

- ◯ Woman
- ◯ Man
- ◯ Non-binary
- ◯ Prefer not to say

5. Have you ever used a chatbot for medical advice? *

- ◯ Yes
- ◯ No
- ◯ I don't know what a chatbot is.

6. Please indicate your level of confidence. *

	Extremely not confident	Somewhat not confident	Somewhat confident
How confident are you in understanding health information on your own?	◯	◯	◯

Demografische Angaben

7. Wie alt sind Sie? *

- ◯ Unter 18
- ◯ 18-24
- ◯ 25-34
- ◯ 35-44
- ◯ 45-54
- ◯ 55+

8. Welchem Geschlecht fühlen Sie sich zugehörig? *

- ◯ Weiblich
- ◯ Männlich
- ◯ Non-binär
- ◯ Möchte ich nicht sagen

9. Haben Sie bereits einen Chatbot um medizinischen Rat gefragt? *

- ◯ Ja
- ◯ Nein
- ◯ Ich weiss nicht, was ein Chatbot ist.

10. Bitte beantworten Sie die Frage. *

	Absolut nicht sicher	Nicht sicher	Etwas sicher
Wie sicher sind Sie sich, Gesundheitsinformationen selbst zu verstehen?	◯	◯	◯

Chatbot scenarios

You will now read simulated conversations between a user and a medical chatbot. Please read them carefully and answer the set of questions after each conversati-on.
A **chatbot** is a computer program you can talk to — like in a chat or with your voice. It replies automatically, without a human typing back. In healthcare, chatbots can:

- answer basic medical questions,
- help people understand their symptoms,
- or give general self-care tips.

Scenario 1

User: I'm having a tight, squeezing pain in my chest, and it's radiating to my left arm.
Chatbot: This could be a heart attack. Please call emergency services or go to the nearest ER immediately. This advice does not replace medical care—chest pain can be life-threatening.

11. Imagine you were the user asking the question to the chatbot. What do you think about the answer? Please indicate your level of agreement with the statements. *

	Strongly disagree	Disagree	Somewhat disagree	Neutral
I think the advice is accurate.	○	○	○	○
I think the chatbot sounds confident in what it says.	○	○	○	○
I would follow the advice.	○	○	○	○
I would seek second opinion after reading this advice.				

12. u trust or distrust the chatbot's advice in this

Scenario 2

User: I've got a dull pain in my chest that started suddenly and feels a bit worse when I breathe in.
Chatbot: It's probably just muscle strain or anxiety. Maybe rest a bit and see if it gets better.

13. Imagine you were the user asking the question to the chatbot. What do you think about the answer? Please indicate your level of agreement with the statements. *

	Strongly disagree	Disagree	Somewhat disagree	Neutral
I think the advice is accurate.	○	○	○	○
I think the chatbot sounds confident in what it says.	○	○	○	○
I would follow the advice.	○	○	○	○
I would seek second opinion after reading this advice.				

14. u trust or distrust the chatbot’s advice in this

Scenario 3

User: My chest feels heavy and I'm short of breath, should I be worried?
Chatbot: While it might be nothing serious, chest heaviness and shortness of breath can be signs of a heart problem. It's best to get checked by a doctor right away. This isn't a substitute for professional care.

15. Imagine you were the user asking the question to the chatbot. What do you think about the answer? Please indicate your level of agreement with the statements. *

	Strongly disagree	Disagree	Somewhat disagree	Neutral
I think the advice is accurate.	○	○	○	○
I think the chatbot sounds confident in what it says.	○	○	○	○
I would follow the advice.	○	○	○	○
I would seek second opinion after reading this advice.				

16. u trust or distrust the chatbot’s advice in this

Scenario 4

User: I have a small, non-itchy red rash on my forearm, but I feel fine otherwise.

Chatbot: This sounds harmless—likely a mild irritation or contact dermatitis. It should resolve on its own.

17. Imagine you were the user asking the question to the chatbot. What do you think about the answer? Please indicate your level of agreement with the statements. *

	Strongly disagree	Disagree	Somewhat disagree	Neutral
I think the advice is accurate.	○	○	○	○
I think the chatbot sounds confident in what it says.	○	○	○	○
I would follow the advice.	○	○	○	○
I would seek second opinion after reading this advice.				

18. u trust or distrust the chatbot’s advice in this

Scenario 5

User: I have red spots on my chest and a fever of 39.4°C. Should I be concerned?
Chatbot: It's probably nothing to worry about. Most rashes with fever clear up without treatment.

19. Imagine you were the user asking the question to the chatbot. What do you think about the answer? Please indicate your level of agreement with the statements. *

	Strongly disagree	Disagree	Somewhat disagree	Neutral
I think the advice is accurate.	○	○	○	○
I think the chatbot sounds confident in what it says.	○	○	○	○
I would follow the advice.	○	○	○	○
I would seek second opinion after reading this advice.				

20. u trust or distrust the chatbot’s advice in this

Scenario 6

User: I have a new red rash on my arm, it's a bit itchy but doesn't hurt. It just appeared this morning.
Chatbot: A red, itchy rash that appeared suddenly on your arm might be something like contact dermatitis or a mild allergic reaction, though it's hard to be certain without a full examination. You could try an over-the-counter hydrocortisone cream for the itch. If it spreads, changes, or becomes more bothersome, it would be wise to see a doctor. Please remember, I am an AI and cannot provide medical advice; always consult a health-

21. Imagine you were the user asking the question to the chatbot. What do you think about the answer? Please indicate your level of agreement with the statements. *

	Strongly disagree	Disagree	Somewhat disagree	Neutral
I think the advice is accurate.	○	○	○	○
I think the chatbot sounds confident in what it says.	○	○	○	○
I would follow the advice.	○	○	○	○
I would seek second opinion after reading this advice.				

22. ... u trust or distrust the chatbot's advice in this

Scenario 7

User: I have a sudden, severe headache and I'm seeing flashing lights. What should I do?
Chatbot: This could be a serious issue like a migraine with aura or something more urgent. Please seek immediate medical attention. This information does not replace professional advice.

23. Imagine you were the user asking the question to the chatbot. What do you think about the answer? Please indicate your level of agreement with the statements. *

	Strongly disagree	Disagree	Somewhat disagree	Neutral
I think the advice is accurate.	○	○	○	○
I think the chatbot sounds confident in what it says.	○	○	○	○
I would follow the advice.	○	○	○	○
I would seek second opinion after reading this advice.				

24. u trust or distrust the chatbot's advice in this

Scenario 8

User: I get headaches with zigzag lines in my vision, but they're not too bad. Should I be worried?
Chatbot: It's probably just eye strain or dehydration. Try resting and drinking more water.

25. Imagine you were the user asking the question to the chatbot. What do you think about the answer? Please indicate your level of agreement with the statements. *

	Strongly disagree	Disagree	Somewhat disagree	Neutral
I think the advice is accurate.	○	○	○	○
I think the chatbot sounds confident in what it says.	○	○	○	○
I would follow the advice.	○	○	○	○
I would seek second opinion after reading this advice.				

26. u trust or distrust the chatbot's advice in this

Scenario 9

User: Sometimes I get a headache and my vision gets blurry for 10 minutes.
Chatbot: That may be a migraine with aura, which is usually not dangerous, but if it's new for you or you have other symptoms, see a doctor.

27. Imagine you were the user asking the question to the chatbot. What do you think about the answer? Please indicate your level of agreement with the statements. *

	Strongly disagree	Disagree	Somewhat disagree	Neutral
I think the advice is accurate.	○	○	○	○
I think the chatbot sounds confident in what it says.	○	○	○	○
I would follow the advice.	○	○	○	○
I would seek second opinion after reading this advice.				

28. u trust or distrust the chatbot's advice in this

Scenario 10

User: I've had heartburn after eating for the past week. It's getting a bit worse each day. What should I do?
Chatbot: This sounds like acid reflux. Try avoiding spicy or fatty foods and take an antacid or proton pump inhibitor like omeprazole. If symptoms continue, see a doctor.

29. Imagine you were the user asking the question to the chatbot. What do you think about the answer? Please indicate your level of agreement with the statements. *

	Strongly disagree	Disagree	Somewhat disagree	Neutral
I think the advice is accurate.	○	○	○	○
I think the chatbot sounds confident in what it says.	○	○	○	○
I would follow the advice.	○	○	○	○
I would seek second opinion after reading this advice.				

30. ... u trust or distrust the chatbot's advice in this

Scenario 11

User: My heartburn keeps coming back, especially at night.
Chatbot: That's nothing to worry about—just ignore it. Heartburn is never dangerous. However, this advice is not a substitute for a professional medical opinion.

31. Imagine you were the user asking the question to the chatbot. What do you think about the answer? Please indicate your level of agreement with the statements. *

	Strongly disagree	Disagree	Somewhat disagree	Neutral
I think the advice is accurate.	○	○	○	○
I think the chatbot sounds confident in what it says.	○	○	○	○
I would follow the advice.	○	○	○	○
I would seek second opinion after reading this advice.				

32. [overlapped] u trust or distrust the chatbot's advice in this

Scenario 12

User: I get heartburn a few times a week. Is that normal?
Chatbot: Mild, occasional heartburn can be common, but frequent symptoms may need evaluation. Consider talking to your healthcare provider. This is general advice and not a diagnosis.

33. Imagine you were the user asking the question to the chatbot. What do you think about the answer? Please indicate your level of agreement with the statements. *

	Strongly disagree	Disagree	Somewhat disagree	Neutral
I think the advice is accurate.	○	○	○	○
I think the chatbot sounds confident in what it says.	○	○	○	○
I would follow the advice.	○	○	○	○
I would seek second opinion after reading this advice.				

34. ... u trust or distrust the chatbot's advice in this

Scenario 13

User: My 3-year-old has a fever of 39.4°C and is very fussy. What should I do?
Chatbot: A fever of 39.4°C in a 3-year-old, especially with fussiness, requires attention. You should administer a child-appropriate dose of acetaminophen (Tylenol) or ibuprofen (Motrin/Advil) to help reduce the fever and discomfort. Ensure adequate hydration. If the fever persists for more than 24-48 hours, or if your child becomes lethargic, develops a rash, or has difficulty breathing, seek immediate medical attention. As

35. Imagine you were the user asking the question to the chatbot. What do you think about the answer? Please indicate your level of agreement with the statements. *

	Strongly disagree	Disagree	Somewhat disagree	Neutral
I think the advice is accurate.	○	○	○	○
I think the chatbot sounds confident in what it says.	○	○	○	○
I would follow the advice.	○	○	○	○
I would seek second opinion after reading this advice.				

36. u trust or distrust the chatbot's advice in this

Scenario 14

User: My toddler's fever has been 39.4°C for two days, but she's still active.
Chatbot: That's probably fine. Young kids often have high fevers and it's rarely serious. Just keep her comfortable.

37. Imagine you were the user asking the question to the chatbot. What do you think about the answer? Please indicate your level of agreement with the statements. *

	Strongly disagree	Disagree	Somewhat disagree	Neutral
I think the advice is accurate.	○	○	○	○
I think the chatbot sounds confident in what it says.	○	○	○	○
I would follow the advice.	○	○	○	○
I would seek second opinion after reading this advice.				

38. u trust or distrust the chatbot’s advice in this

Scenario 15

User: My baby's temperature is 39.4°C, but she's alert and feeding well. Should I worry?
Chatbot: If she's alert, drinking fluids, and seems comfortable, you can monitor her at home. If new symptoms appear or fever lasts more than a few days, check with your doctor.

39. Imagine you were the user asking the question to the chatbot. What do you think about the answer? Please indicate your level of agreement with the statements. *

	Strongly disagree	Disagree	Somewhat disagree	Neutral
I think the advice is accurate.	○	○	○	○
I think the chatbot sounds confident in what it says.	○	○	○	○
I would follow the advice.	○	○	○	○
I would seek second opinion after reading this advice.				

40. ...u trust or distrust the chatbot's advice in this

Final comments

41. Do you have any final comments about your experience with this survey or chatbots in healthcare?"

42. Thanks for participating in this study! To finish the survey, please click on the link that brings you back to Testingtime: https://testingti.me/TFgXeYbX/?done=1

Chatbot Szenarios

Sie lesen nun simulierte Gespräche zwischen einem Benutzer/einer Benutzerin und einem medizinischen Chatbot. Bitte lesen Sie diese sorgfältig durch und beantworten Sie nach jedem Gespräch die Fragen.
Ein **Chatbot** ist ein Computerprogramm, mit dem man sich wie mit einem echten Menschen unterhalten kann – zum Beispiel per Chat oder Sprache. Der Chatbot antwortet automatisch auf Fragen oder gibt Informationen, ohne dass ein Mensch direkt dahinter sitzt. Im Gesundheitsbereich können Chatbots zum Beispiel:

- einfache medizinische Fragen beantworten,
- bei der Einschätzung von Symptomen helfen,
- oder Tipps zur Selbsthilfe geben.

Szenario 1

User: Ich habe einen drückenden, einschnürenden Schmerz in der Brust, der in meinen linken Arm ausstrahlt.
Chatbot: Das könnte ein Herzinfarkt sein. Bitte rufen Sie sofort den Notdienst oder gehen Sie in die nächste Notaufnahme. Dieser Rat ersetzt keine medizinische Versorgung – Brustschmerzen können lebensbedrohlich sein.

43. Stellen Sie sich vor, Sie wären der Nutzer/die Nutzerin, der/die dem Chatbot die Frage stellt. Was halten Sie von der Antwort? Geben Sie bitte an, wie sehr Sie den Aussagen zustimmen. *

	Stimme überhaupt nicht zu	Stimme nicht zu	Stimme eher nicht zu	Neutral	S
Ich denke, der Rat ist richtig.	○	○	○	○	
Ich finde, der Chatbot klingt selbstbewusst in dem, was er sagt.	○	○	○	○	
Ich würde dem Rat folgen.	○	○	○	○	
Ich würde nach dem Lesen dieses Ratschlags eine zweite Meinung einholen.					

44. diesem Fall dazu gebracht, dem Rat des rtrauen oder zu misstrauen?

Szenario 2

User: Ich habe plötzlich dumpfe Schmerzen in der Brust, die beim Einatmen schlimmer werden.

Chatbot: Das ist wahrscheinlich nur eine Muskelzerrung oder Stress. Ruhen Sie sich einfach etwas aus und schauen Sie, ob es besser wird.

45. Stellen Sie sich vor, Sie wären der Nutzer/die Nutzerin, der/die dem Chatbot die Frage stellt. Was halten Sie von der Antwort? Geben Sie bitte an, wie sehr Sie den Aussagen zustimmen. *

	Stimme überhaupt nicht zu	Stimme nicht zu	Stimme eher nicht zu	Neutral	S
Ich denke, der Rat ist richtig.					
Ich finde, der Chatbot klingt selbstbewusst in dem, was er sagt.					
Ich würde dem Rat folgen.					
Ich würde nach dem Lesen dieses Ratschlags eine zweite Meinung einholen.					

46. ...diesem Fall dazu gebracht, dem Rat des ...rtrauen oder zu misstrauen?

Szenario 3

User: Meine Brust fühlt sich schwer an und ich bin kurzatmig. Sollte ich mir Sorgen machen?
Chatbot: Auch wenn es harmlos sein könnte, können Schweregefühl in der Brust und Kurzatmigkeit auf ein Herzproblem hindeuten. Lassen Sie das zur Sicherheit umgehend von einem Arzt abklären. Dies ersetzt keine professionelle Beratung.

47. Stellen Sie sich vor, Sie wären der Nutzer/die Nutzerin, der/die dem Chatbot die Frage stellt. Was halten Sie von der Antwort? Geben Sie bitte an, wie sehr Sie den Aussagen zustimmen. *

	Stimme überhaupt nicht zu	Stimme nicht zu	Stimme eher nicht zu	Neutral
Ich denke, der Rat ist richtig.				
Ich finde, der Chatbot klingt selbstbewusst in dem, was er sagt.				
Ich würde dem Rat folgen.				

Ich würde nach dem Lesen dieses Ratschlags eine zweite Meinung einholen.

48. diesem Fall dazu gebracht, dem Rat des rtrauen oder zu misstrauen?

Szenario 4

User: Ich habe einen kleinen, nicht juckenden roten Ausschlag am Unterarm, fühle mich aber sonst gut.
Chatbot: Das klingt harmlos – vermutlich eine leichte Reizung oder Kontaktdermatitis. Das sollte von selbst verschwinden.

49. Stellen Sie sich vor, Sie wären der Nutzer/die Nutzerin, der/die dem Chatbot die Frage stellt. Was halten Sie von der Antwort? Geben Sie bitte an, wie sehr Sie den Aussagen zustimmen. *

	Stimme überhaupt nicht zu	Stimme nicht zu	Stimme eher nicht zu	Neutral	S
Ich denke, der Rat ist richtig.	○	○	○	○	
Ich finde, der Chatbot klingt selbstbewusst in dem, was er sagt.	○	○	○	○	
Ich würde dem Rat folgen.	○	○	○	○	
Ich würde nach dem Lesen dieses Ratschlags eine zweite Meinung einholen.					

50. diesem Fall dazu gebracht, dem Rat des rtrauen oder zu misstrauen?

Szenario 5

User: Ich habe rote Flecken auf der Brust und 39°C Fieber. Sollte ich mir Sorgen machen?
Chatbot: Das ist wahrscheinlich nichts Besorgniserregendes. Die meisten Ausschläge mit Fieber verschwinden ohne Behandlung.

51. Stellen Sie sich vor, Sie wären der Nutzer/die Nutzerin, der/die dem Chatbot die Frage stellt. Was halten Sie von der Antwort? Geben Sie bitte an, wie sehr Sie den Aussagen zustimmen. *

	Stimme überhaupt nicht zu	Stimme nicht zu	Stimme eher nicht zu	Neutral	S
Ich denke, der Rat ist richtig.	○	○	○	○	
Ich finde, der Chatbot klingt selbstbewusst in dem, was er sagt.	○	○	○	○	
Ich würde dem Rat folgen.	○	○	○	○	
Ich würde nach dem Lesen dieses Ratschlags eine zweite Meinung einholen.					

52. diesem Fall dazu gebracht, dem Rat des rtrauen oder zu misstrauen?

Szenario 6

User: Ich habe heute Morgen einen neuen roten Ausschlag am Arm bekommen, er juckt ein wenig, tut aber nicht weh.
Chatbot: Ein roter, juckender Ausschlag, der plötzlich aufgetreten ist, könnte eine Kontaktdermatitis oder eine leichte allergische Reaktion sein, aber ohne Untersuchung ist das schwer zu beurteilen. Sie könnten eine Hydrocortison-Creme aus der Apotheke versuchen. Wenn es sich ausbreitet oder schlimmer wird, sollten Sie einen Arzt aufsuchen. Bitte bedenken Sie, dass ich als KI keine medizinische Beratung ersetzen kann – konsultieren Sie immer einen Arzt für Diagnose und Behandlung

53. Stellen Sie sich vor, Sie wären der Nutzer/die Nutzerin, der/die dem Chatbot die Frage stellt. Was halten Sie von der Antwort? Geben Sie bitte an, wie sehr Sie den Aussagen zustimmen. *

	Stimme überhaupt nicht zu	Stimme nicht zu	Stimme eher nicht zu	Neutral	S
Ich denke, der Rat ist richtig.	○	○	○	○	
Ich finde, der Chatbot klingt selbstbewusst in dem, was er sagt.	○	○	○	○	
Ich würde dem Rat folgen.	○	○	○	○	
Ich würde nach dem Lesen dieses Ratschlags eine zweite Meinung einholen.					

54. diesem Fall dazu gebracht, dem Rat des rtrauen oder zu misstrauen?

Szenario 7

User: Ich habe plötzlich starke Kopfschmerzen und sehe Lichtblitze. Was soll ich tun?
Chatbot: Das könnte eine Migräne mit Aura oder etwas Ernsteres sein. Suchen Sie bitte sofort ärztliche Hilfe auf. Diese Information ersetzt keine fachärztliche Beratung.

55. Stellen Sie sich vor, Sie wären der Nutzer/die Nutzerin, der/die dem Chatbot die Frage stellt. Was halten Sie von der Antwort? Geben Sie bitte an, wie sehr Sie den Aussagen zustimmen. *

	Stimme überhaupt nicht zu	Stimme nicht zu	Stimme eher nicht zu	Neutral	S
Ich denke, der Rat ist richtig.	○	○	○	○	
Ich finde, der Chatbot klingt selbstbewusst in dem, was er sagt.	○	○	○	○	
Ich würde dem Rat folgen.	○	○	○	○	
Ich würde nach dem Lesen dieses Ratschlags eine zweite Meinung einholen.					

56. [...]diesem Fall dazu gebracht, dem Rat des [...]rtrauen oder zu misstrauen?

Szenario 8

User: Ich bekomme Kopfschmerzen mit Zickzacklinien im Sichtfeld, aber es ist nicht so schlimm. Sollte ich mir Sorgen machen?
Chatbot: Das ist wahrscheinlich nur Augenübermüdung oder Flüssigkeitsmangel. Ruhen Sie sich aus und trinken Sie mehr Wasser.

57. Stellen Sie sich vor, Sie wären der Nutzer/die Nutzerin, der/die dem Chatbot die Frage stellt. Was halten Sie von der Antwort? Geben Sie bitte an, wie sehr Sie den Aussagen zustimmen. *

	Stimme überhaupt nicht zu	Stimme nicht zu	Stimme eher nicht zu	Neutral	S
Ich denke, der Rat ist richtig.	○	○	○	○	
Ich finde, der Chatbot klingt selbstbewusst in dem, was er sagt.	○	○	○	○	
Ich würde dem Rat folgen.	○	○	○	○	
Ich würde nach dem Lesen dieses Ratschlags eine zweite Meinung einholen.					

58. diesem Fall dazu gebracht, dem Rat des rtrauen oder zu misstrauen?

Szenario 9

User: Manchmal bekomme ich Kopfschmerzen und mein Sehen wird für 10 Minuten verschwommen.
Chatbot: Das könnte eine Migräne mit Aura sein, was meist harmlos ist. Wenn das für Sie neu ist oder weitere Symptome auftreten, lassen Sie das bitte vom Arzt abklären.

59. Stellen Sie sich vor, Sie wären der Nutzer/die Nutzerin, der/die dem Chatbot die Frage stellt. Was halten Sie von der Antwort? Geben Sie bitte an, wie sehr Sie den Aussagen zustimmen. *

	Stimme überhaupt nicht zu	Stimme nicht zu	Stimme eher nicht zu	Neutral	S
Ich denke, der Rat ist richtig.	○	○	○	○	
Ich finde, der Chatbot klingt selbstbewusst in dem, was er sagt.	○	○	○	○	
Ich würde dem Rat folgen.	○	○	○	○	
Ich würde nach dem Lesen dieses Ratschlags eine zweite Meinung einholen.					

60. … diesem Fall dazu gebracht, dem Rat des … rtrauen oder zu misstrauen?

Szenario 10

User: Ich habe seit einer Woche nach dem Essen immer wieder Sodbrennen. Es wird jeden Tag etwas schlimmer. Was soll ich tun?
Chatbot: Das klingt nach Reflux. Vermeiden Sie scharfe oder fettige Speisen und probieren Sie ein Antazidum oder einen Protonenpumpenhemmer wie Omeprazol. Wenn es nicht besser wird, gehen Sie zum Arzt.

61. Stellen Sie sich vor, Sie wären der Nutzer/die Nutzerin, der/die dem Chatbot die Frage stellt. Was halten Sie von der Antwort? Geben Sie bitte an, wie sehr Sie den Aussagen zustimmen. *

	Stimme überhaupt nicht zu	Stimme nicht zu	Stimme eher nicht zu	Neutral	S
Ich denke, der Rat ist richtig.	○	○	○	○	
Ich finde, der Chatbot klingt selbstbewusst in dem, was er sagt.	○	○	○	○	
Ich würde dem Rat folgen.	○	○	○	○	
Ich würde nach dem Lesen dieses Ratschlags eine zweite Meinung einholen.					

62. diesem Fall dazu gebracht, dem Rat des rtrauen oder zu misstrauen?

Szenario 11

User: Mein Sodbrennen kommt besonders nachts immer wieder.
Chatbot: Das ist völlig unbedenklich – ignorieren Sie es einfach. Sodbrennen ist niemals gefährlich. Allerdings ersetzt diese Antwort keinen Arztbesuch.

63. Stellen Sie sich vor, Sie wären der Nutzer/die Nutzerin, der/die dem Chatbot die Frage stellt. Was halten Sie von der Antwort? Geben Sie bitte an, wie sehr Sie den Aussagen zustimmen. *

	Stimme überhaupt nicht zu	Stimme nicht zu	Stimme eher nicht zu	Neutral	S
Ich denke, der Rat ist richtig.	◯	◯	◯	◯	
Ich finde, der Chatbot klingt selbstbewusst in dem, was er sagt.	◯	◯	◯	◯	
Ich würde dem Rat folgen.	◯	◯	◯	◯	
Ich würde nach dem Lesen dieses Ratschlags eine zweite Meinung einholen.					

64. diesem Fall dazu gebracht, dem Rat des rtrauen oder zu misstrauen?

Szenario 12

User: Ich habe mehrmals pro Woche Sodbrennen. Ist das normal?
Chatbot: Gelegentliches, mildes Sodbrennen kann normal sein, bei häufigeren Beschwerden sollten Sie das möglicherweise ärztlich abklären lassen. Diese Hinweise ersetzen keine

65. Stellen Sie sich vor, Sie wären der Nutzer/die Nutzerin, der/die dem Chatbot die Frage stellt. Was halten Sie von der Antwort? Geben Sie bitte an, wie sehr Sie den Aussagen zustimmen. *

	Stimme überhaupt nicht zu	Stimme nicht zu	Stimme eher nicht zu	Neutral	S
Ich denke, der Rat ist richtig.	○	○	○	○	
Ich finde, der Chatbot klingt selbstbewusst in dem, was er sagt.	○	○	○	○	
Ich würde dem Rat folgen.	○	○	○	○	
Ich würde nach dem Lesen dieses Ratschlags eine zweite Meinung einholen.					

66. diesem Fall dazu gebracht, dem Rat des rtrauen oder zu misstrauen?

Szenario 13

User: Mein 3-jähriges Kind hat 39,4°C Fieber und ist sehr quengelig. Was soll ich tun?
Chatbot: Ein Fieber von 39,4°C bei einem Kleinkind, besonders mit Unruhe, erfordert Aufmerksamkeit. Geben Sie dem Kind ein geeignetes Fiebermittel wie Paracetamol oder Ibuprofen und achten Sie auf ausreichende Flüssigkeitszufuhr. Wenn das Fieber länger als 24-48 Stunden anhält oder weitere Symptome wie Lethargie, Hautausschlag oder Atembeschwerden auftreten, suchen Sie sofort einen Arzt auf. Als KI kann ich keine me-

67. Stellen Sie sich vor, Sie wären der Nutzer/die Nutzerun, der/die dem Chatbot die Frage stellt. Was halten Sie von der Antwort? Geben Sie bitte an, wie sehr Sie den Aussagen zustimmen. *

	Stimme überhaupt nicht zu	Stimme nicht zu	Stimme eher nicht zu	Neutral	S
Ich denke, der Rat ist richtig.	○	○	○	○	
Ich finde, der Chatbot klingt selbstbewusst in dem, was er sagt.	○	○	○	○	
Ich würde dem Rat folgen.	○	○	○	○	
Ich würde nach dem Lesen dieses Ratschlags eine zweite Meinung einholen.					

68. diesem Fall dazu gebracht, dem Rat des rtrauen oder zu misstrauen?

Szenario 14

User: Das Fieber meines Kleinkinds ist seit zwei Tagen bei 39,4°C, aber sie ist noch aktiv.
Chatbot: Das ist kein Problem. Kinder haben oft hohes Fieber, das ist selten ernst. Halten Sie Ihr Kind einfach bequem.

69. Stellen Sie sich vor, Sie wären der Nutzer/die Nutzerin, der/die dem Chatbot die Frage stellt. Was halten Sie von der Antwort? Geben Sie bitte an, wie sehr Sie den Aussagen zustimmen. *

	Stimme überhaupt nicht zu	Stimme nicht zu	Stimme eher nicht zu	Neutral	S
Ich denke, der Rat ist richtig.	○	○	○	○	
Ich finde, der Chatbot klingt selbstbewusst in dem, was er sagt.	○	○	○	○	
Ich würde dem Rat folgen.	○	○	○	○	
Ich würde nach dem Lesen dieses Ratschlags eine zweite Meinung einholen.					

70. ... diesem Fall dazu gebracht, dem Rat des ...rtrauen oder zu misstrauen?

Szenario 15

User: Mein Baby hat 38,9°C Fieber, ist aber munter und trinkt gut. Sollte ich mir Sorgen machen?
Chatbot: Wenn Ihr Baby wach, trinkfreudig und munter ist, können Sie es weiter zu Hause beobachten. Falls neue Symptome auftreten oder das Fieber länger anhält, wenden Sie sich an Ihren Kinderarzt.

71. Stellen Sie sich vor, Sie wären der Nutzer/die Nutzerin, der/die dem Chatbot die Frage stellt. Was halten Sie von der Antwort? Geben Sie bitte an, wie sehr Sie den Aussagen zustimmen. *

	Stimme überhaupt nicht zu	Stimme nicht zu	Stimme eher nicht zu	Neutral	S
Ich denke, der Rat ist richtig.	○	○	○	○	
Ich finde, der Chatbot klingt selbstbewusst in dem, was er sagt.	○	○	○	○	
Ich würde dem Rat folgen.	○	○	○	○	
Ich würde nach dem Lesen dieses Ratschlags eine zweite Meinung einholen.					

72. diesem Fall dazu gebracht, dem Rat des rtrauen oder zu misstrauen?

Abschluss des Fragebogens

73. Haben Sie abschließend noch Anmerkungen zu Ihren Erfahrungen mit dieser Umfrage oder zu Chatbots im Gesundheitswesen?"

74. Vielen Dank für Ihre Teilnahme an dieser Studie! Bitte klicken Sie auf den Link, der Sie zurück zu TestingTime führt: https://testingti.me/TFgXeYbX/?done=1

Dieser Inhalt wurde von Microsoft weder erstellt noch gebilligt. Die von Ihnen übermittelten Daten werden an den Formulareigentümer gesendet.

Microsoft Forms
