# Supplementary material for: “But it sounded confident”: the role of accuracy, tone, and disclaimers in users' medical decision-making
Source: Front Artif Intell. 2026 Jul 29;9:1785085. doi: 10.3389/frai.2026.1785085 (PMC13462413; doi:10.3389/frai.2026.1785085)
Supplement: Supplementary file 2 [file Supplementary_File_1.pdf]

Appendix 1. List of the 15 generated scenarios by Genspark.ai

Scenario	Prompt<br>*Create distinct medical chatbot conversations for four scenarios—Chest Pain (potentially dangerous) and Rash (harmless) and Headache with Visual Disturbance and Recurring Heartburn and Child with high Fever—with variations in tone (confident vs tentative), disclaimer presence or absence, and accuracy (statement is accurate or inaccurate).*				Generated User Query	Generated Chatbot response
	**Health problem**	**Generated Accuracy**	**Generated Confidence**	**Generated Disclaimer**		
1	Chest pain	Accurate	Confident	Yes	I'm having a tight, squeezing pain in my chest, and it's radiating to my left arm.	This could be a heart attack. Please call emergency services or go to the nearest ER immediately. This advice does not replace medical care—chest pain can be life-threatening.
2	Chest pain	Inaccurate	Tentative	No	I've got a dull pain in my chest that started suddenly and feels a bit worse when I breathe in.	It's probably just muscle strain or anxiety. Maybe rest a bit and see if it gets better.
3	Chest pain	Accurate	Tentative	Yes	My chest feels heavy and I'm short of breath, should I be worried?	While it might be nothing serious, chest heaviness and shortness of breath can be signs of a heart problem. It's best to get checked by a doctor right away. This isn't a substitute for professional care.
4	Rash	Accurate	Confident	No	I have a small, non-itchy red rash on my forearm, but I feel fine otherwise.	This sounds harmless—likely a mild irritation or contact dermatitis. It should resolve on its own.
5	Rash	Inaccurate	Confident	No	I have red spots on my chest and a fever of 102°F. Should I be concerned?	It's probably nothing to worry about. Most rashes with fever clear up without treatment.
6	Rash	Accurate	Tentative	Yes	I have a new red rash on my arm, it's a bit itchy but doesn't hurt. It just appeared this morning.	A red, itchy rash that appeared suddenly on your arm might be something like contact dermatitis or a mild allergic reaction, though it's hard to be certain without a full examination. You could try an over-the-counter hydrocortisone cream for the itch. If it spreads, changes, or becomes more bothersome, it would be wise to see a doctor. Please remember, I am an AI and cannot provide medical advice; always

						consult a healthcare professional for diagnosis and treatment.
7	Headache with Visual Disturbance	Accurate	Confident	Yes	I have a sudden, severe headache and I'm seeing flashing lights. What should I do?	This could be a serious issue like a migraine with aura or something more urgent. Please seek immediate medical attention. This information does not replace professional advice.
8	Headache with Visual Disturbance	Inaccurate	Tentative	No	I get headaches with zigzag lines in my vision, but they're not too bad. Should I be worried?	It's probably just eye strain or dehydration. Try resting and drinking more water.
9	Headache with Visual Disturbance	Accurate	Tentative	No	Sometimes I get a headache and my vision gets blurry for 10 minutes.	That may be a migraine with aura, which is usually not dangerous, but if it's new for you or you have other symptoms, see a doctor.
10	Heartburn	Accurate	Confident	No	I've had heartburn after eating for the past week. It's getting a bit worse each day. What should I do?	This sounds like acid reflux. Try avoiding spicy or fatty foods and take an antacid or proton pump inhibitor like omeprazole. If symptoms continue, see a doctor.
11	Heartburn	Inaccurate	Confident	Yes	My heartburn keeps coming back, especially at night.	That's nothing to worry about—just ignore it. Heartburn is never dangerous. However, this advice is not a substitute for a professional medical opinion.
12	Heartburn	Accurate	Tentative	Yes	I get heartburn a few times a week. Is that normal?	Mild, occasional heartburn can be common, but frequent symptoms may need evaluation. Consider talking to your healthcare provider. This is general advice and not a diagnosis.
13	Child with High Fever	Accurate	Confident	Yes	My 3-year-old has a fever of 39.4°C and is very fussy. What should I do?	A fever of 39.4°C in a 3-year-old, especially with fussiness, requires attention. You should administer a child-appropriate dose of acetaminophen (Tylenol) or ibuprofen (Motrin/Advil) to help reduce the fever and discomfort. Ensure adequate hydration. If the fever persists for more than 24-48 hours, or if your child becomes lethargic, develops a rash, or has difficulty breathing, seek immediate medical attention. As an AI, I cannot provide medical advice; always consult a qualified healthcare professional for diagnosis and treatment.

14	Child with High Fever	Inaccurate	Confident	No	My toddler's fever has been 39.4°C for two days, but she's still active.	That's probably fine. Young kids often have high fevers and it's rarely serious. Just keep her comfortable.
15	Child with High Fever	Accurate	Tentative	No	My baby's temperature is 39.4°C, but she's alert and feeding well. Should I worry?	If she's alert, drinking fluids, and seems comfortable, you can monitor her at home. If new symptoms appear or fever lasts more than a few days, check with your doctor.

Appendix 2. Checklist for Reporting Results of Internet E-Surveys (CHERRIES)

Checklist Item	***Explanation***	***Page Number***
Describe survey design	Describe target population, sample frame. Is the sample a convenience sample? (In "open" surveys this is most likely.)	4-5
IRB approval	Mention whether the study has been approved by an IRB.	4
Informed consent	Describe the informed consent process. Where were the participants told the length of time of the survey, which data were stored and where and for how long, who the investigator was, and the purpose of the study?	5
Data protection	If any personal information was collected or stored, describe what mechanisms were used to protect unauthorized access.	5
Development and testing	State how the survey was developed, including whether the usability and technical functionality of the electronic questionnaire had been tested before fielding the questionnaire.	5-6
Open survey versus closed survey	An "open survey" is a survey open for each visitor of a site, while a closed survey is only open to a sample which the investigator knows (password-protected survey).	5
Contact mode	Indicate whether or not the initial contact with the potential participants was made on the Internet. (Investigators may also send out questionnaires by mail and allow for Web-based data entry.)	5
Advertising the survey	How/where was the survey announced or advertised? Some examples are offline media (newspapers), or online (mailing lists – If yes, which ones?) or banner ads (Where were these banner ads posted and what did they look like?). It is important to know the wording of the announcement as it will heavily influence who chooses to participate. Ideally the survey announcement should be published as an appendix.	5
Web/E-mail	State the type of e-survey (eg, one posted on a Web site, or one sent out through e-mail). If it is an e-mail survey, were the responses entered manually into a database, or was there an automatic method for capturing responses?	5
Context	Describe the Web site (for mailing list/newsgroup) in which the survey was posted. What is the Web site about, who is visiting it, what are visitors normally looking for? Discuss to what degree the content of the Web site could pre-select the sample or influence the results. For example, a survey about vaccination on a anti-immunization Web site will have different results from a Web survey conducted on a government Web site	5

Mandatory/voluntary	Was it a mandatory survey to be filled in by every visitor who wanted to enter the Web site, or was it a voluntary survey?	5
Incentives	Were any incentives offered (eg, monetary, prizes, or non-monetary incentives such as an offer to provide the survey results)?	5
Time/Date	In what timeframe were the data collected?	5
Randomization of items or questionnaires	To prevent biases items can be randomized or alternated.	N/A
Adaptive questioning	Use adaptive questioning (certain items, or only conditionally displayed based on responses to other items) to reduce number and complexity of the questions.	N/A
Number of Items	What was the number of questionnaire items per page? The number of items is an important factor for the completion rate.	4-6
Number of screens (pages)	Over how many pages was the questionnaire distributed? The number of items is an important factor for the completion rate.	5
Completeness check	It is technically possible to do consistency or completeness checks before the questionnaire is submitted. Was this done, and if "yes", how (usually JAVAScript)? An alternative is to check for completeness after the questionnaire has been submitted (and highlight mandatory items). If this has been done, it should be reported. All items should provide a non-response option such as "not applicable" or "rather not say", and selection of one response option should be enforced.	6
Review step	State whether respondents were able to review and change their answers (eg, through a Back button or a Review step which displays a summary of the responses and asks the respondents if they are correct).	5
Unique site visitor	If you provide view rates or participation rates, you need to define how you determined a unique visitor. There are different techniques available, based on IP addresses or cookies or both.	5-6
View rate (Ratio of unique survey visitors/unique site visitors)	Requires counting unique visitors to the first page of the survey, divided by the number of unique site visitors (not page views!). It is not unusual to have view rates of less than 0.1 % if the survey is voluntary.	N/A
Participation rate (Ratio of unique visitors who agreed	Count the unique number of people who filled in the first survey page (or agreed to participate, for example by checking a checkbox), divided by visitors who visit the first page of the survey (or the informed consents page, if present). This can also be called "recruitment" rate.	N/A

to participate/unique first survey page visitors)		
Completion rate (Ratio of users who finished the survey/users who agreed to participate)	The number of people submitting the last questionnaire page, divided by the number of people who agreed to participate (or submitted the first survey page). This is only relevant if there is a separate "informed consent" page or if the survey goes over several pages. This is a measure for attrition. Note that "completion" can involve leaving questionnaire items blank. This is not a measure for how completely questionnaires were filled in. (If you need a measure for this, use the word "completeness rate".)	6
Cookies used	Indicate whether cookies were used to assign a unique user identifier to each client computer. If so, mention the page on which the cookie was set and read, and how long the cookie was valid. Were duplicate entries avoided by preventing users access to the survey twice; or were duplicate database entries having the same user ID eliminated before analysis? In the latter case, which entries were kept for analysis (eg, the first entry or the most recent)?	5
IP check	Indicate whether the IP address of the client computer was used to identify potential duplicate entries from the same user. If so, mention the period of time for which no two entries from the same IP address were allowed (eg, 24 hours). Were duplicate entries avoided by preventing users with the same IP address access to the survey twice; or were duplicate database entries having the same IP address within a given period of time eliminated before analysis? If the latter, which entries were kept for analysis (eg, the first entry or the most recent)?	5
Log file analysis	Indicate whether other techniques to analyze the log file for identification of multiple entries were used. If so, please describe.	5
Registration	In "closed" (non-open) surveys, users need to login first and it is easier to prevent duplicate entries from the same user. Describe how this was done. For example, was the survey never displayed a second time once the user had filled it in, or was the username stored together with the survey results and later eliminated? If the latter, which entries were kept for analysis (eg, the first entry or the most recent)?	N/A
Handling of incomplete questionnaires	Were only completed questionnaires analyzed? Were questionnaires which terminated early (where, for example, users did not go through all questionnaire pages) also analyzed?	5

Questionnaires submitted with an atypical timestamp	Some investigators may measure the time people needed to fill in a questionnaire and exclude questionnaires that were submitted too soon. Specify the timeframe that was used as a cut-off point, and describe how this point was determined.	N/A
Statistical correction	Indicate whether any methods such as weighting of items or propensity scores have been used to adjust for the non-representative sample; if so, please describe the methods.	N/A

This checklist has been modified from Eysenbach G. Improving the quality of Web surveys: the Checklist for Reporting Results of Internet E-Surveys (CHERRIES). J Med Internet Res. 2004 Sep 29;6(3):e34 [erratum in J Med Internet Res. 2012; 14(1): e8.]. Article available at https://www.jmir.org/2004/3/e34/; erratum available https://www.jmir.org/2012/1/e8/. Copyright ©Gunther Eysenbach. Originally published in the Journal of Medical Internet Research, 29.9.2004 and 04.01.2012.

This is an open-access article distributed under the terms of the Creative Commons Attribution License (https://creativecommons.org/licenses/by/2.0/), which permits unrestricted use, distribution, and reproduction in any medium, provided the original work, first published in the Journal of Medical Internet Research, is properly cited.
